# Change Partners, Regrow an Axon

**DOI:** 10.1371/journal.pbio.1001919

**Published:** 2014-08-05

**Authors:** Richard Robinson

**Affiliations:** Freelance Science Writer, Sherborn, Massachusetts, United States of America

Axons rarely regrow after a severe spinal cord injury, in part because of inhibitory signals associated with myelin, which surrounds and insulates the axon. These signals bind to the Nogo receptor, which can then bind to a variety of co-receptors, including a protein called p75. Together, this complex triggers a cascade of intracellular signals that ultimately inhibits axonal sprouting and prevents regeneration.

Understanding how p75 is regulated, therefore, may shed light on new strategies for promoting recovery from nerve damage. In this issue of *PLOS Biology*, Marçal Vilar, Tsung-Chang Sung, and Kuo-Fen Lee show that p75 may lose its affinity for the Nogo receptor through interaction with a second protein, whose structure is closely related to p75 itself.

**Figure 1:**
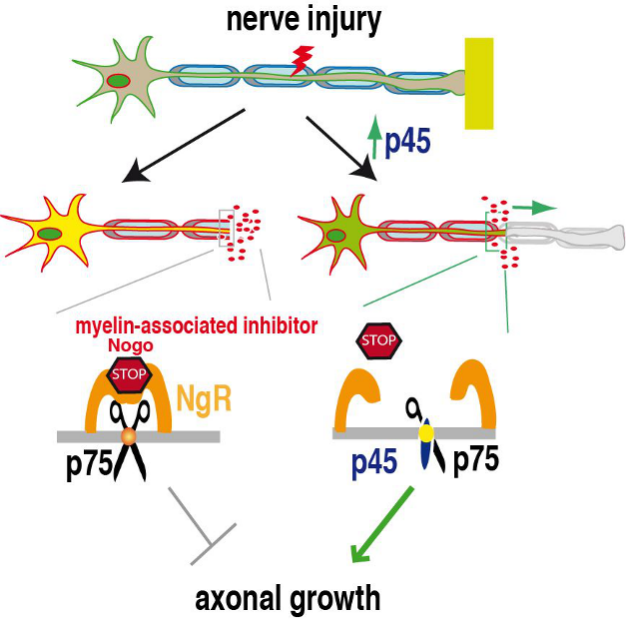
Following nerve injury, increasing p45 levels in neurons promotes nerve growth by blocking the formation of p75 homodimers that are critical for transmitting inhibitory signals from myelin-associated inhibitors such as Nogo.

p75 bears an intracellular region (the chillingly named “death domain”) which, when it occurs in many related proteins, allows the protein bearing it to associate with others like it and to trigger signaling. One such protein is p45, and so the authors asked whether p45 and p75 might interact. In extracts from mouse cerebellum, they found that, indeed, the two proteins formed a complex and that p45's expression pattern in response to injury matched that of p75. Increasing the expression of p45, they noticed, reduced the association of p75 and the Nogo receptor, an effect dependent on both intracellular and transmembrane domains of p45, indicating the likely site of physical association between the two proteins (Figure 1).

To confirm that hypothesis, the authors next characterized in detail the intracellular domain of p75. They found that in a gel, purified p75 displayed two different molecular weights, one twice that of the other, suggesting it forms a homodimer. Next, they systematically mutated specific amino acids throughout the protein and used nuclear magnetic resonance spectroscopy to map the effect on binding. As they suspected, the death domain played a key role in binding the two monomers together. A covalent cysteine linkage between the two further stabilized the homodimer. That stability contributed to the dimer's ability to bind to the Nogo receptor.

Next, they used similar techniques to characterize p45's intracellular regions and to show that p45 linked to p75 through the death domains on each. By changing the concentrations of the two in solution, they found that p45 could disrupt the homodimer, leading to formation of a p45–p75 heterodimer, an effect that depended in part on cysteine–cysteine interactions within the transmembrane domains of each.

Finally, they showed that the disruption of the p75 homodimer, along with the formation of the p45–p75 heterodimer, reduced the association of p75 with the Nogo receptor and, therefore, reduced the complex's ability to trigger the signaling that inhibits axon growth, thereby promoting axonal regrowth.

While mice express p45, humans do not, because of a stop codon mutation, raising the question of whether this regulatory system is absent in humans, or whether other, as-yet-unidentified proteins play a similar role in regulating the p75–Nogo receptor interaction. In either case, the ability to disrupt that interaction, either directly or by targeting those putative proteins, may offer a new avenue for spinal cord repair.

**Vilar M, Sung T-C, Chen Z, García-Carpio I, Fernandez EM, et al. (2014) Heterodimerization of p45–p75 Modulates p75 Signaling: Structural Basis and Mechanism of Action.** doi:10.1371/journal.pbio.1001918

